# Descriptive Study and Surgical Management Among Infiltrating Lobular Carcinoma Patients Admitted to King Abdulaziz Medical City From 2000 to 2017: A Retrospective Cross-Sectional Study

**DOI:** 10.7759/cureus.35180

**Published:** 2023-02-19

**Authors:** Sara I Alshahwan, Ghada Alsowailmi, Amal Alotaibi, Afnan Alsahli, Mosaad I Alshahwan, Amal Alasfoor, Aamir Omair, Lolwah A Alriyees

**Affiliations:** 1 Medicine and Surgery, National Guard Health Affairs, Riyadh, SAU; 2 Medicine and Surgery, National Guard hospital Affairs, Riyadh, SAU; 3 Medicine and Surgery, King Saud Bin Abdulaziz University for Health Sciences College of Medicine, Riyadh, SAU; 4 Nursing, National Guard Health Affairs, Riyadh, SAU; 5 Biostatistics and Epidemiology, King Saud Bin Abdulaziz University for Health Sciences College of Medicine, Riyadh, SAU; 6 General Surgery, King Abdulaziz Medical City Riyadh, Riyadh, SAU

**Keywords:** metastasis, mammogram, modified radical mastectomy, biopsy, invasive lobular breast carcinoma

## Abstract

Introduction: Invasive lobular carcinoma (ILC) is the second most common histologic type of breast carcinoma. The etiology of ILC is unknown; however, many contributing risk factors have been suggested. Treatment of ILC can be divided into local and systemic. Our objectives were to assess the clinical presentations, risk factors, radiological findings, pathological types, and surgical options for patients with ILC treated at the national guard hospital. Identify the factors associated with metastasis and recurrence.

Methods: Retrospective cross-sectional descriptive study at a tertiary care center in Riyadh. All adult patients aged 16 years and above, from different nationalities, and both genders, were diagnosed with ILC from 2000 to 2017 and followed up at KAMC. The sampling technique was a non-probability consecutive technique. Among 1066 patients identified, 91 patients were diagnosed with ILC over seventeen years study period.

Results: The median age at the primary diagnosis was 50. On the clinical examination, 63 (71%) cases were found to have palpable masses which was the most suspicious finding. On radiology, the most encountered finding was speculated masses which were seen in 76 (84%). Regarding the pathology, unilateral breast cancer was seen in 82 while bilateral breast cancer was found only in eight. For the biopsy, a core needle biopsy was the most commonly used in 83 (91%) patients. The most documented surgery for ILC patients was a modified radical mastectomy. Metastasis in different organs was identified with the musculoskeletal system being the commonest site. Different significant variables were compared between patients with or without metastasis. Skin changes, post-operative invasion, estrogen, progesterone, and HER2 receptors were significantly associated with metastasis. Patients with metastasis were less likely to have conservative surgery. Regarding the Recurrence and five years survival, out of 62 cases, 10 had recurrence within five years, which was more prevalent in patients who had fine needle aspiration, excisional biopsy, and nulliparous patients.

Conclusion: To our knowledge, this is the first study to exclusively describe ILC in Saudi Arabia. The results of this current study are highly important, as these results provide baseline data of ILC in the capital city of Saudi Arabia.

## Introduction

Invasive lobular carcinoma (ILC) is the second most common histologic type of breast carcinoma, with an incidence of 5.8%, as reported by the Saudi National Cancer Registry [1]. ILC is a distinct malignant neoplasm that consists of small cells arranged in loose cohesive structures within the lobules. The cells individually invade the stroma, forming an “Indian file” between the collagen bundles [2]. ILC is frequently multicentric and bilateral and expresses hormone receptors with rare overexpression of HER2/NEU receptors [2].

The etiology of ILC is unknown; however, many contributing risk factors have been suggested, including female sex, increasing age, ethnic origin, positive family history, genetic predisposition, estrogen exposure, radiation exposure, increased breast density, alcohol consumption, obesity, and a sedentary lifestyle [3]. Evaluating these factors enables high-risk individuals to get regular screening and determine weighted cost-benefit options, including watchful waiting, prophylactic oophorectomy or mastectomy, and chemoprevention [3].

Most ILC patients present with a palpable mass or mammographic changes, yet a significant group is clinically occult despite the diffuse invasive pattern [2]. ILC detection by mammography can be challenging, as the lesions' opacity tends to be equal to or less than that of the parenchyma. Given this, radiologists should be highly suspicious of any abnormal mammographic findings. Previous studies investigated the mammographic findings in ILC and observed the following: spiculated opacity (53%), architectural distortion (16%), poorly defined opacity (7%), normal or benign findings (16%), and parenchymal asymmetry (4%) [4]. Conversely, ultrasound is more sensitive than mammography in ILC detection [5]. Paramagul et al. investigated the sonographic characteristics of ILC and found that 13 out of 19 patients had ultrasound changes. Masses with irregular margins, heterogeneous internal echoes, and acoustic attenuation were noted in seven patients, and ultrasound findings mimicking benign lesions were noted in six patients [6].

On biopsy, the classic cells of ILC are of two types: type A cells (classic) or larger type B cells with vesicular nuclei with mild pleomorphism [7]. In classic ILC, the infiltrating cells invade the basement membrane as single files of cells, creating the characteristic targetoid growth pattern. Other morphological variants have also been observed, including pleomorphic, alveolar, solid, and mixed ductal-lobular variants [7]. Pleomorphic lobular carcinoma (PLC) is characterized by cellular atypia and marked nuclear pleomorphism. PLC may also have an increased mitotic rate, signet ring cells, or mucinous or histiocytoid morphology. The alveolar and solid variants are characterized by classic ILC cells aligned in aggregates of at least 20 cells (alveolar variant) or sheets (solid variant). Furthermore, mixed ductal-lobular variation is exhibited in 5% of all invasive breast carcinomas [7].

The treatment of ILC can be divided into local (surgical resection and radiation) and systemic (endocrine therapy and chemotherapy) based on the stage and grade of the tumor. Due to the difficulty in estimating the extent of infiltrative growth of ILC, more aggressive surgeries, including mastectomy and axillary lymph node dissection (ALND), have been favored [8]. However, with screening and the significant decrease in the size of tumors at times of diagnosis, breast conservative therapy (BCT), consisting of segmental mastectomy and radiation therapy, has been more widely accepted. In fact, several clinical trials have shown that BCT is equally effective as standard mastectomy for early-stage ILC [8]. A study that included 248 patients with ILC showed that the local recurrence rate in 36 women who underwent BCT was equal to the 212 women treated by mastectomy [9].

Classical ILC generally responds well to endocrine therapy due to the special phenotype of low histological grade, low mitotic index, positive hormone receptors, negative HER2, P53, and basal markers [7]. In comparison, the poor prognosis was documented in pleomorphic ILC, a less common and more aggressive phenotype of ILC. Pleomorphic ILC can lose hormone receptor expression, demonstrate HER2/NEU amplification, and tend to be larger with increased metastasis [10]. Despite the favorable biologic phenotype of ILC, patients do not have a better clinical outcome compared to invasive ductal carcinoma (IDL), as the overall five-year survival rate is 85.6% and 84.1%, respectively [11].

As ILC is less prevalent than infiltrating ductal carcinoma (IDC), descriptive outlines of ILC have not been adequately reported in the literature. This indicates that more studies need to be carried out to understand the nature of this serious disease. Moreover, the recurrence rate in patients who underwent a mastectomy compared to other types of surgery is still controversial and needs to be assessed. The aim of the study is to assess the clinical presentations, risk factors, radiological findings, pathological types, and surgical options for patients with ILC treated at the National Guard Hospital and identify the factors associated with metastasis and recurrence.

## Materials and methods

This study design was a cross-sectional study conducted in King Abdulaziz Medical City KAMC, Riyadh, Saudi Arabia. The general surgery department is one of the biggest departments in KAMC. The breast clinic provides services to more than 2,500 patients yearly. In particular, there are 150-180 breast cancer patients per year.

The study population was adult patients aged 16 years and above, from different nationalities and both genders, and who were diagnosed with ILC from 2000 to 2017 and followed up at KAMC. No exclusion criteria were used. The sample size included every patient who fulfilled the criteria. The sampling technique was a non-probability consecutive technique since all the cases were included. ILC was diagnosed on the suggestive history, compatible physical findings, radiological findings, and pathological results.

Approval of the research submission was received from the institutional review board (IRB); the protocol number is RC19/081/R. The data did not contain any personal information about the participants. A retrospective chart review was conducted in which the investigators reviewed paper and electronic charts to extract the data. Variables included were patients' demographics (age, gender, and nationality), risk factors (menopause, age of menarche, age at full-term pregnancy, hormonal replacement therapy, and family history of breast cancer), investigation (radiological findings and pathological results), and management (local and systemic). The independent variables were age, gender, nationality, and past reproductive history. The outcome variables were the recurrence rate and the development of metastasis.

Data entry was managed using MS Excel, and the data were analyzed using the Statistical Package for the Social Sciences (SPSS). The results are presented as descriptive statistics, which are presented as the mean and standard deviation for numerical variables (e.g., age) and as frequencies and percentages for the categorical variables (e.g., gender, nationality, and risk factor status). A chi-square test was used to compare the categorical variables. The data are presented with a 95% confidence interval, and a P-value < 0.05 was statistically significant.

## Results

Among 1,066 patients identified in the database with different breast lesions, 91 were admitted with ILC over a 17-year study period. The median age at primary diagnosis was 50 years (range 34-88). The demographics, risk factors, and clinical presentations of the patients are summarized in Table 1. On clinical examination, 63 (71%) cases were found to have palpable masses, which was the most suspicious finding. Skin changes were observed in 32 (36%) patients, and nipple discharge was found in four (4.5%) patients (Table 1).

**Table 1:** Patients demographics (N=91)

Patients demographics	N (%)
Female	91 (100%)
Saudi	79 (87%)
Married	83 (95%)
Pre-menopause	41 (49%)
Post-menopause	42 (51%)
Menarche<13	19 (31%)
Age at full-term delivery>30	3 (5%)
Nulliparous	6 (7%)
Oral contraceptives	28 (31%)
Hormonal replacement therapy	8 (9%)
Prior breast biopsy	13 (15%)
Family history of breast cancer	22 (25%)
Clinical presentations	
Breast lump	63 (71%)
Skin changes	32 (36%)
Nipple discharge	4 (4.5%)

In Table 2, radiological abnormalities in mammography and ultrasound were described in the majority of the patients. These findings consist of 76 (84%) speculated masses, 38 (42%) radiological microcalcifications, and 33 (37%) cases of stromal distortion. Breast Imaging-Reporting and Data System (BIRADS) was used with BIRADS 1 seen in only one patient. BIRADS 2 was not identified. BIRADS 3 was observed in six (7%) patients. BIRADS 4 was seen in 18 (20%). BIRADS 5 was the most documented finding in 61 (68.5%) patients. There were three (3%) cases of BIRADS 6. The tumor characteristics are shown in Table 2.

**Table 2:** Radiological and pathological tumor characteristics (N=91) ER: Estrogen receptor, PR: Progesterone receptor, HER2: Human epidermal growth factor receptor 2

Tumor characteristics	N (%)
Radiological findings	
Radiological microcalcification	38 (42%)
Radiological mass	76 (84%)
Radiological stromal distortion	33 (37%)
Breast Imaging - Reporting and Data System (BI-RADS)	
1	1 (1%)
2	0 (0%)
3	6 (7%)
4	18 (20%)
5	61 (69%)
6	3 (3%)
Unilateral breast cancer	82 (91%)
Bilateral breast cancer	8 (9%)
Quadrant	
Upper outer quadrant	50 (56%)
Upper inner quadrant	9 (10%)
Lower outer quadrant	11 (12%)
Lower inner quadrant	9 (10%)
Retro-areolar region	8 (9%)
Biopsy	
Core needle biopsy	83 (91%)
Fine needle aspiration	1 (1%)
Excisional biopsy	7 (8%)
Grade	
Low	30 (34%)
Medium	53 (60%)
High	6 (7%)
Necrosis	10 (11%)
Microinvasion	16 (18%)
Microcalcification	13 (14%)
Post-operative invasion	7 (8%)
Axillary lymph node involvement	41 (45%)
ER positivity	84 (92%)
PR positivity	71 (78%)
Human epidermal growth factor receptor 2 (HER2)	11 (12%)

Regarding the pathology, unilateral breast cancer was seen in 82 (91%) cases, while bilateral breast cancer was found only in eight (9%). The most common location was the upper outer quadrant (UOQ), with 50 (56%) cases, followed by the lower outer quadrant (LOQ) in 11 (12%), the upper inner quadrant (UIQ) in nine (10%), the lower inner quadrant (LIQ) in nine (10%), and retro-areolar in only eight (9%). For the biopsy, a core needle biopsy was the most commonly used in 83 (91%) patients, while an excisional biopsy and a fine needle aspiration were performed in seven (8%) and one (1%) patients, respectively. Different pathological grades were detected, with the medium grade being the highest at 53 (60%), followed by the low grade at 30 (34%), and the high grade being the lowest at six (7%). Other pathological abnormalities were described, including necrosis in 10 (11%), microinvasion in 16 (18%), macrocalcification in 13 (14%), and postoperative invasion in seven (8%). Most of the patients with ILC tended to have positive estrogen receptors (ER) in 84 (92%), followed by positive progesterone receptors (PR) in 71 (78%), with a few cases of positive human epidermal growth factor receptors 2 (HER2) in 11 (12%). Axillary lymph node involvement tends to be positive in nearly half of the patients (45%) (Table 2).

In the surgical management, as summarized in Table 3, 35 (38.5%) of ILC patients underwent a modified radical mastectomy, 17 (19%) cases had breast-conserving therapy, 13 (14%) had a mastectomy, and six patients had an oncoplastic and mastectomy with reconstruction. On the other hand, we found that 20 (22%) patients did not undergo any surgery, and 27 (30%) underwent palliative therapy. A sentinel lymph node biopsy was done in 36 patients, the results of which were positive in 15 and negative in 21 patients. The majority of patients, 50 (55%), did not have SLN and underwent axillary clearance immediately 50 (55%). Systemic management included hormonal therapy in 79 (87%) patients, followed by chemotherapy in 58 (64%) and radiotherapy in 58 (64%). Recurrence was described in 10 patients (11%).

**Table 3:** Patients’ Management (N=91)

Patients’ Management	N (%)
Surgical options	
Not done	20 (22%)
Breast conservative therapy	17 (19%)
Oncoplastic	3 (3%)
Mastectomy with reconstruction	3 (3%)
Mastectomy	13 (14%)
Modified radical mastectomy	35 (39%)
Sentinel lymph node	
Not done	55 (60%)
Negative	21 (23%)
Positive	15 (17%)
Axillary clearance	50 (55%)
Management (Systemic)	
Chemotherapy	58 (64%)
Hormonal therapy	79 (87%)
Radiotherapy	58 (64%)
Palliative therapy	27 (30%)
Recurrence	10 (11%)
Metastasis	38 (42%)
Respiratory system	16 (18%)
Gastrointestinal system	22 (24%)
Reproductive system	7 (8%)
Nervous system	9 (10%)
Musculoskeletal system	25 (28%)
Lymph node	24 (26%)
Endocrine system	1 (1%)

Metastasis to different organs was identified with the musculoskeletal system being the most common site for distant metastasis in 25 (27.5%) cases, followed by lymph node involvement in 24 (26%), the gastrointestinal system in 22 (24%), the respiratory system in 16 (18%), the nervous system in nine (10%), the reproductive system in seven (8%), and the endocrine system being the least common site for metastasis in only one patient. The median age for follow-up was seven, with one year being the lowest and 21 years being the highest (Table 3).

Different significant variables were compared between patients with or without metastasis, as shown in Table 4. Skin changes were more common (51%) in patients with metastasis compared to 25% of patients without metastasis (P=0.11). A positive family history of breast cancer was found in only 11% of patients with metastasis compared to 35% of patients without metastasis (P=0.01). The majority of patients with or without metastasis had unilateral breast cancer (81% and 98%, respectively). Conversely, patients with metastasis were more likely to have bilateral lesions (19%) as compared to those with no metastasis (2%) (P=0.008). The postoperative invasion was rare in patients without metastasis (2%) compared to patients with metastasis (16%) (P=0.20). Estrogen and progesterone receptors were also significantly associated with metastasis. Moreover, among patients with metastasis, 86% had positive ER, and 68% had positive PR, as compared to 98% positive ER and 88% positive PR in patients without metastasis (P=0.008 and 0.016, respectively). Patients with metastasis were more likely to be HER2 positive (19%) compared to patients with no metastasis (8%) (P=0.09). Given the high number of positive receptors on pathology, 79% of patients with metastasis and 92% without metastasis were on hormonal therapy (P=0.06). There was a significant difference (P<0.001) in the surgical options applied to patients with metastasis and no metastasis. Patients with metastasis were less likely to have conservative surgery (5%) compared to 30% of those with no metastasis. Surgery was not done in almost half (47%) of the patients with metastasis, compared to 4% in those without metastasis. A mastectomy with or without reconstruction was done in 24% of patients without metastasis and 11% with metastasis. A modified radical mastectomy was done in 37% of patients with metastasis and 42% without metastasis. Interestingly, there was a significant difference between BMI and metastasis. Metastasis was less likely to develop in obese patients. Out of all patients with BMI less than 30, 57% had metastasis, while 43% of patients with a BMI equal to or above 30 developed metastasis (P=0.002) (Table 4). 

**Table 4:** Association between metastasis and different variables * p-value measured by the Fisher exact test ** Missing one from the Metastasis group ‡ Missing two from the Metastasis group and one from the No metastasis group

Variables	Total	With Metastasis (N=38)	Without Metastasis (N=53)	p-value (Chi-square test)
Skin changes	32	19 (51%)	13 (25%)	0.011
Family history of breast cancer	22	4(11%)	18(35%)	0.010
Tumor’s location^**^				
Unilateral	82	30(81%)	52(98%)	0.008^*^
Bilateral	8	7(19%)	1(2%)	0.008^*^
Postoperative invasion	7	6(16%)	1(2%)	0.02^*^
Estrogen receptor (ER)	84	32(86%)	52(98%)	0.08^*^
Progesterone receptor (PR)	71	25(68%)	46(88%)	0.016
Human epidermal growth factor receptor 2 (HER2)	11	7(19%)	4(8%)	0.09
Surgical management ^‡^				
Not done	20	18(47%)	2(4%)	<0.001
Breast conservative therapy	17	2(5%)	15(30%)	<0.001
Mastectomy ±reconstruction	16	4(11%)	12(24%)	<0.001
Modified radical mastectomy	35	14(37%)	21(42%)	<0.001
Hormonal therapy	79	30(79%)	49(92%)	0.06
Body mass index (Kg/m^2^)				
Less than 30	34	21(57%)	13(25%)	0.002
30 and above	56	16(43%)	40(75%)	0.002

**Figure 1:**
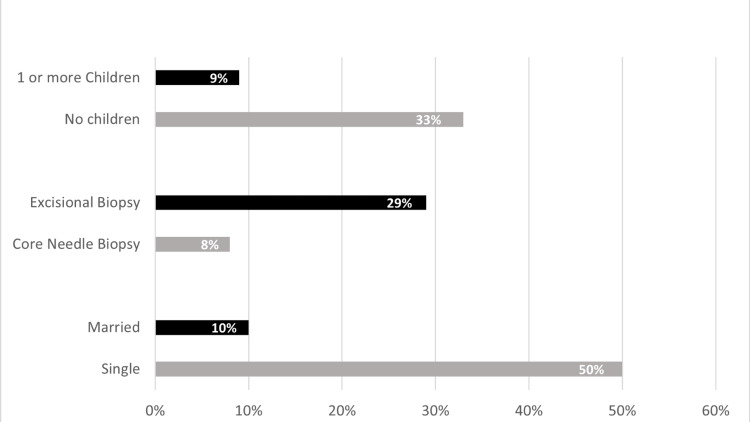
Percentage of patients having recurrence

Patient demographics, risk factors, clinical presentations, radiological findings, pathological results, and management were compared between patients who had a recurrence and those who did not. Of all the variables included, marital status, the number of children, and biopsy were significantly different between the two groups, as shown in Figure 1. For marital status, 50% of single patients and 10% of married patients had a recurrence (P=0.063). There were 33% of nulliparity patients with a recurrence, compared to 9% of those with one or more children (P=0.06). On biopsy, 8% of patients who had a core needle biopsy, 100% of patients who had a fine needle aspiration, and 29% of patients who had an excisional biopsy had a recurrence (P=0.02) (Figure 1).

## Discussion

This study investigated the risk factors, clinical presentations, radiological findings, pathological results, and the management of ILC. ILC is the second most common cancer worldwide. It is influenced by several risk factors. This study showed that patients with ILC are, on average, elderly at presentation, corroborating other studies [11].

The relationship between reproductive factors and the risk of breast cancer is well established in the literature [12-14]. In the current study, we investigated some of these relationships. The most common risk factor encountered was post-menopause status, seen in 51% of our patients. This finding is in good agreement with those of Bernhard et al., in which postmenopausal women accounted for 53% of their sample [15]. Moreover, early menarche was another reproductive factor that Brinton et al. established in association with breast cancer. Their study concluded that women who began menstruating before the age of 12 have a 1.3-fold increased risk of invasive breast cancer compared to those who began after the age of 15 [12]. This established risk is somewhat similar to what we found in our patients in which early menarche before the age of 13 was seen in up to 31% of our patients with a mean age at menarche of 13.2 (1.7) years.

The Brinton studies also revealed that a nulliparous woman has a 1.6-fold increased risk of breast cancer. On the other hand, women giving birth at the age of 30 or older have a 2.2-fold increased risk of breast cancer [12-14]. Their results, however, go against our findings in which nulliparity was seen in 7%, and only 5% of our patients gave birth to their firstborn after the age of 30. The relationship between breast cancer and the aforementioned reproductive factors was explained by Vogel, who suggested that the main contributing factor was the number of ovulatory menstrual cycles that a woman goes through in her lifetime [16]. Moreover, to prove this suggestion, it has been documented that women who have both ovaries removed at an age younger than 40 have a 45% reduction in risk in comparison to those who have natural menopause at the age of 50 to 54 [12].

A positive family history is one of the acknowledged risk factors for ILC. Compared to individuals with a negative family history of breast cancer, Pharoah et al. estimated a 1.8-fold increased risk for a first-degree relative who developed breast cancer at 50 years of age or older and a 3.3-fold increased risk for a first-degree relative who developed breast cancer at an age younger than 50 years [17]. As anticipated in the present study, up to one-quarter of our patients had a positive family history of breast cancer. However, our finding is almost half the result of national data from Jeddah published by Syed et al., in which 44.5% of their sample had a positive family history [18].

In the current study, the rate of oral contraceptive use was 31%; however, higher rates were noticed in the western region of Saudi Arabia (58%) [18]. There are conflicting data regarding whether the use of oral contraceptives is considered a risk factor for breast cancer. A meta-analysis failed to prove such an association, with a relative risk of 1.10 [19]. While a smaller study in Saudi Arabia has also demonstrated this lack of association overall, they found a correlation between the use of oral contraceptives for more than 10 years and breast cancer risk [18]. However, given their small sample size, the conclusion needs to be confirmed by a more extensive study.

Obesity has been linked to breast cancer. In the present study, the prevalence of patients with a BMI of 30 or more was 62%, almost similar to that of a national study that found that 75.8% of breast cancer patients had abnormal weight. Moreover, they concluded that obese women manifest a more than two-fold greater breast cancer risk than those with normal body mass index [20]. It is not surprising that high BMI is at least a minor risk factor for breast cancer because adipose tissue is an essential extragonadal source of bio-available estrogens [21,22].

Our results are consistent with the findings of Veronica et al., who found that 82% of the patients had a palpable mass and the most common mammographic findings associated with ILC were the presence of speculated masses in 39% of the patients and architectural distortion in 34% of the patients. Mammography has detected microcalcifications associated with ILC in only 2%. Our study showed microcalcifications in up to 42% of cases. This difference likely reflects the fact that we included all histologic subtypes of ILC [5].

Several studies have reported a high incidence of contralateral tumors in ILC patients [9,23]. This is contrary to our results, in which only 9% of the patients had bilateral breast cancer. This could be explained by the fact that the majority of these studies compared the risk of developing contralateral breast lesions between IDC and ILC. Other variables could increase the risk of bilaterality, such as the intensity of lymphoid infiltrate, tumor markers, blood type, and age at diagnosis. Moreover, the decline in the current study could be attributed to the earlier detection of breast tumors, improved treatment techniques, and extended follow-up. The median follow-up in our study was seven years. Our results confirmed that UOQ of the breast was the anatomical location of greatest involvement, followed by LOQ and UIQ, corroborating data from other studies [9,24]. However, retro-areolar was the second most frequent localization, while in our study, it was a rare finding [9]. In this study, like others [7,11,25], ILC tumors were found to be of medium to low histological grade, hormone receptors positive, HER2 negative, and possessing a low rate of microvasion.

BCT is considered a better surgical option than MRM because it provides equivalent local control with significantly better cosmetic results. Our results found that patients were treated more often with MRM than BCT, which is similar to results from other studies [26,27]. This could be attributed to the difficulty in defining the margins of the tumor clinically, histologically, and radiologically in ILC, as it tends to be multicentric or multifocal and larger; moreover, this decision is not only influenced by the pathological findings but also by patients’ preferences. In addition, our study included all patients from 2000 to 2017, so we must consider the changing trends in surgical modalities. Concerning systemic therapy, since ILC is more frequently hormonal receptor-positive, as expected, a greater portion of our patients received hormonal therapy [11].

Concerning the pattern of metastatic spread in ILC, several studies observed an increased incidence of bone, distant lymph node, gastrointestinal, and gynecological metastasis, and a lower incidence of spreading to the lung and nervous system [11,17]. Our study is in accordance with these reports except for reproductive metastasis, which was only found in 7% of the cases. The increased tendency toward bone metastasis in ILC patients could be related to the status of the hormonal receptor.

In the present study, different variables, including patient demographics, risk factors, clinical presentations, radiological findings, pathological results, and management, were compared with metastasis and recurrence in order to evaluate if there was any association. Of these comparisons, several associations were found to be significant in this study. However, after thoroughly reviewing the literature, only one of our significant associations has been studied in the literature. In the current study, metastasis was less likely to develop in obese patients. However, our finding contradicts what is documented in three studies that have found three different mechanisms through which obesity promotes metastasis [28-30]. Moreover, in another study that compared the median overall survival (MOS) with BMI, they concluded that women with a normal BMI had a MOS of 54 months, whereas, in women with an overweight BMI, this number decreased to 28 months [31].

In this study, some limitations need to be recognized. First, our study was limited by its small sample size. Second, the study design was a retrospective cross-sectional study. Third, the study was conducted at a single hospital in the capital city of Saudi Arabia and may not be generalizable to the entire country. Finally, we could not obtain detailed data regarding reproductive history, investigations, and treatments because these pieces of information were not written in the medical records, or the patients were transferred between hospitals and medical records at hospitals other than the sample hospitals. However, the current study has many strengths. First, we included all adult patients aged 16 and above who were diagnosed with ILC without having any other exclusion criteria. Second is the long-term extension of this study as we included all ILC patients for a 17-year period.

## Conclusions

To our knowledge, this is the first study to describe ILC exclusively in Saudi Arabia. The results of this current study are significant, as these results provide baseline data of ILC in the capital city of Saudi Arabia, thus contributing to future epidemiological and hospital-based research. We recommend further epidemiological and descriptive studies in different regions of Saudi Arabia, along with educational campaigns and screening programs, to address this problem.
